# HSPA4 Is a Biomarker of Placenta Accreta and Enhances the Angiogenesis Ability of Vessel Endothelial Cells

**DOI:** 10.3390/ijms23105682

**Published:** 2022-05-19

**Authors:** Sung-Chou Li, Kuo-Chung Lan, Hsuan-Ning Hung, Wan-Ting Huang, Yun-Ju Lai, Hsin-Hsin Cheng, Chih-Chang Tsai, Kun-Long Huang, Huey-Ling You, Te-Yao Hsu

**Affiliations:** 1 Center for Mitochondrial Research and Medicine and Genomics and Proteomics Core Laboratory, Kaohsiung Chang Gung Memorial Hospital and Chang Gung University College of Medicine, Kaohsiung 833, Taiwan; raymond.pinus@gmail.com; 2 Department of Obstetrics and Gynecology, Kaohsiung Chang Gung Memorial Hospital and Chang Gung University College of Medicine, Kaohsiung 833, Taiwan; lankuochung@gmail.com (K.-C.L.); agneshenrysean@gmail.com (H.-N.H.); lusionbear@cgmh.org.tw (Y.-J.L.); chokovarous@cgmh.org.tw (H.-H.C.); aniki@cgmh.org.tw (C.-C.T.); mr9221@cgmh.org.tw (K.-L.H.); 3 Department of Obstetrics and Gynecology, Jen-Ai Hospital, Taichung 412, Taiwan; 4 Department of Laboratory Medicine, Kaohsiung Chang Gung Memorial Hospital and Chang Gung University College of Medicine, Kaohsiung 833, Taiwan; huangwanting5@gmail.com (W.-T.H.); youhling@cgmh.org.tw (H.-L.Y.)

**Keywords:** placenta accreta, proteomics, HSPA4, angiogenesis, placenta invasion, biomarker

## Abstract

Placenta accreta spectrum (PAS) accounts for 7% of maternal mortality and is associated with intraoperative and postoperative morbidity caused by massive blood loss, infection, and adjacent organ damage. The aims of this study were to identify the protein biomarkers of PAS and to further explore their pathogenetic roles in PAS. For this purpose, we collected five placentas from pregnant subjects with PAS complications and another five placentas from normal pregnancy (NP) cases. Then, we enriched protein samples by specifically isolating the trophoblast villous, deeply invading into the uterine muscle layer in the PAS patients. Next, fluorescence-based two-dimensional difference gel electrophoresis (2D-DIGE) and MALDI-TOF/MS were used to identify the proteins differentially abundant between PAS and NP placenta tissues. As a result, nineteen spots were determined as differentially abundant proteins, ten and nine of which were more abundant in PAS and NP placenta tissues, respectively. Then, specific validation with western blot assay and immunohisto/cytochemistry (IHC) assay confirmed that heat shock 70 kDa protein 4 (HSPA4) and chorionic somatomammotropin hormone (CSH) were PAS protein biomarkers. Further tube formation assays demonstrated that HSPA4 promoted the in vitro angiogenesis ability of vessel endothelial cells, which is consistent with the in vivo scenario of PAS complications. In this study, we not only identified PAS protein biomarkers but also connected the promoted angiogenesis with placenta invasion, investigating the pathogenetic mechanism of PAS.

## 1. Introduction

In recent decades, the incidence rate of the placenta accreta spectrum (PAS) has risen from 1 in 4027 to 1 in 533 deliveries in the US due to the increased number of patients undergoing cesarean sections [1]. According to the invasion depth of chorionic villi, abnormal invasive placenta is classified into accreta, increta, and percreta. PAS is a potential lethal cause of obstetric hemorrhage [2]. Although the pathogenesis of abnormal placentation is not yet clearly understood, the most accepted theory is that it results from an imbalance between decidualization and trophoblast invasion. Dysfunctional decidua in a scarred area caused by previous uterine surgery leads to invasion of the myometrium by the placenta [3].

Prior uterine surgery, myomectomy, and cesarean section were all reported to be associated with PAS [4]. Other risk factors included maternal age over 35 years, grandmultiparity and low socioeconomic status [5]. In addition, preeclampsia was also reported to be highly associated with PAS. PAS accounts for a 7% maternal mortality rate and is associated with intraoperative and postoperative mortality caused by either massive blood transfusion, infection, or adjacent organ damage [1]. In practice, a prenatal combination of grayscale, color Doppler ultrasonography, [6] and MRI is used to establish the diagnosis, but it is limited [7].

The pathogenesis mechanisms of PAS are complicated and multifactorial. The differentiation, proliferation, and invasion of trophoblast cells require a sophisticated regulatory mechanism involving the following elements: growth factors, receptors, cell-adhesion molecules, extracellular matrix proteins, hormones, and transcription factors [3]. Decidual defects are still a major contributing factor to the formation of placenta accreta. Trophoblasts, the specialized cells of the placenta, play a major role in the implantation and formation of the maternal-fetal interface. In recent years, a great deal of knowledge has been gained about the regulatory mechanisms, from transcriptional networks to oxygen tension, which control trophoblast differentiation. Calcitonin and MAPK are associated with trophoblast penetration of endometrial epithelium [8]. MMP, integrins and oxygen are associated with trophoblast invasion of endometrial stroma [9]. Some studies also reported several PAS-associated proteins, including vascular endothelial growth factor (VEGF), placenta growth factor (PlGF), their receptors (VEGFR), epidermal growth factor receptor, c-erbB-2 oncoprotein, angiopoietin-1, angiopoietin-2, and Tie receptors [10,11]. Therefore, extensive neovascularization is clearly evident in the majority of PAS cases. However, to our knowledge, there is no model to evaluate angiogenesis in the placenta of PAS.

The goal of this study is to identify the potential protein biomarkers of PAS and to investigate their pathogenetic mechanism on PAS. Comparative proteomic assays combined with bioinformatics tools are widely used for the detection of disease biomarkers [12,13]. To date, however, no mass spectrometry has been applied for placenta implantation, especially for PAS.

## 2. Results

### 2.1. The Characteristics of the Patients with Placenta Accreta and the Subjects with Normal Pregnancy

In this study, we enrolled ten pregnant subjects to donate their placenta tissues after delivery. Among the ten subjects, five were normal pregnancy (NP) cases and the other five were the cases with PAS complications. We first compared the NP and PAS subjects in terms of age, gestational age and newborn weight. As shown in Table 1, no significant difference was observed among these factors. Moreover, although not significant, the PAS subjects had a higher mean previous history of uterine surgery (1.4 ± 0.51 vs 0.4 ± 0.4; *p*-value = 0.16). All five PAS subjects underwent hysterectomy (Table 2), among which, two were placenta increta and three were placenta percreta cases. All PAS subjects suffered blood loss of at least 1500 mL. Moreover, case 1 and case 2 of PAS subjects had disseminated intravascular coagulation and were transferred to the intensive care unit for postpartum care. All 10 cases had no preeclamptic history.

### 2.2. Protein Abundance Variations between PAS and NP Placenta Tissues

**Figure 1:**
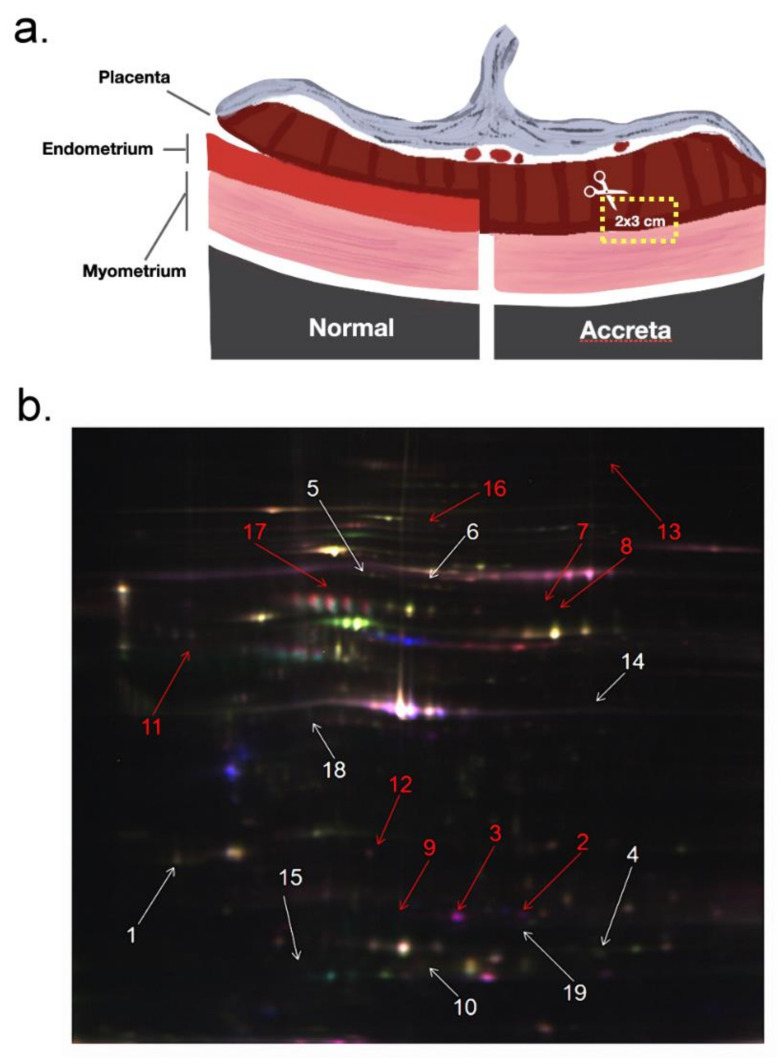
The illustration of the collected tissue sample from placenta trophoblast villous and the example of one 2D-DIGE assay on the collected placental tissue sample. (**a**) For the placenta tissues of PAS subjects, the trophoblast villous invading into the uterine muscle layer was collected. For the placenta tissues of normal pregnant subjects, the common trophoblast villous without invading into the uterine muscle layer was collected. (**b**) Through five independent comparisons (NP vs. PAS), protein quantification can be performed. Arrowheads indicated the spots differentially abundant between NP and PAS placenta tissues. The 19 differentially abundant spots were excised for protein identification by MALDI-TOF.

From the PAS placentas, we collected the trophoblast villous tissues invading into the uterine muscle layer (Figure 1a). Then, the collected tissue blocks were further applied for different assays. We used 2D-DIGE to compare protein abundance variations between NP and PAS placenta tissues. Figure 1b illustrates the results of 2D-DIGE and silver staining assays on one NP and one PAS placenta tissues. As shown in Figure 1b, 19 spots seemed to be differentially abundant between PAS and NP placenta tissues. By five comparisons (5 PAS vs. 5 NP), we calculated the *p*-values and tabulated the details of these 19 spots (determined with MALDI-TOF MS/MS) in Table 3. Table 3 demonstrates that these 19 protein spots kept at least 1.5-fold abundance variations. In addition, ten spots (highlighted by the red arrow) were more abundant in the PAS placenta tissues than in the NP ones, whereas nine spots (highlighted by the white arrow) were less abundant in the PAS placenta tissues.

**Figure 2:**
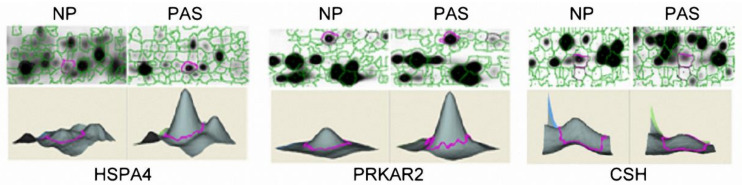
The results of protein quantification with 2D-DIGE. After labeling, the images of gels were scanned in a Typhoon 9400 scanner and further analyzed with Decyder software to quantify protein abundance. HSPA4 and PRKAR2 had higher abundances in PAS tissues, whereas CSH was more abundant in NP tissues.

Among the 19 differentially abundant proteins, we were especially interested in the cAMP-dependent protein kinase type II-alpha regulatory subunit (PRKAR2) and the heat shock 70 kDa protein 4 (HSPA4) since they had the largest positive fold change ratio (PAS vs. NP). In addition, the chorionic somatomammotropin hormone (CSH) was also examined due to its possible functions with placenta development. Figure 2 demonstrated the abundance variations (determined with 2D-DIGE) of the three proteins in one pair of PAS and NP tissues. As shown in Figure 2, HSPA4 (spot 2) and PRKAR2 (spot 3) were circled (upper lane) in the 2D-gel electrophoresis and their abundances were further quantified (lower lane) to illustrate higher levels in PAS placenta tissues. A similar presentation was also conducted on CSH (spot 10) with higher levels in NP placenta tissues.

### 2.3. Validation of the Differentially Abundant Proteins

**Figure 3:**
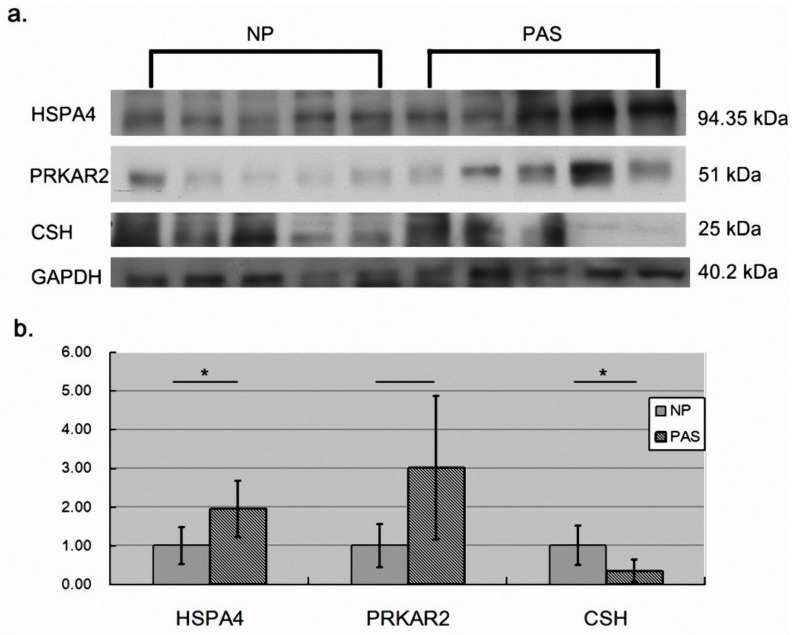
The results of western blot assays. We used western blot assays to validate the variations in protein abundance determined with 2D-DIGE. (**a**) The western blot results. (**b**) With GAPDH as an internal control, HSPA4 and CSH reached statistical significance (*n* = 5). * denoted *p*-value < 0.05 (*t*-test). Data was presented as the mean ± S.D.

**Figure 4:**
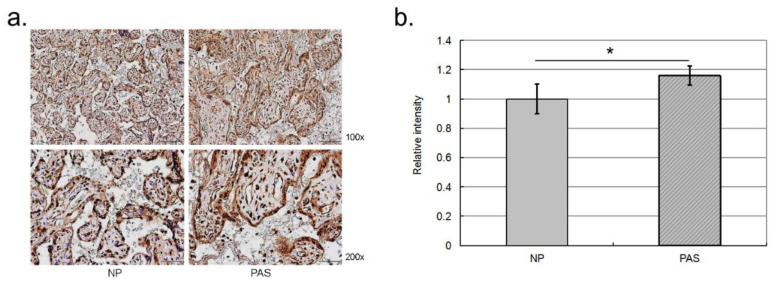
The results of IHC assays. We used IHC assay to examine the abundance of HSPA4 protein among the FFPE trophoblast villous. The scanned images were analyzed with GraphPad Prism 5. (**a**) IHC result on one of the trophoblast villous from one PAS and one normal pregnant subject. (**b**) Quantification result of the placenta samples from five PAS and five normal pregnant subjects. * denoted *p*-value < 0.05.

To provide more solid evidence, we examined the three proteins with western blot assays (Figure 3). Consistent with the 2D-DIGE assay, HSPA4 and PRKAR2 were more abundant in PAS placenta than in NP placenta tissues although PRKAR2 did not reach significance (*p*-value = 0.072). CSH also maintained a significant higher level in NP placenta than in PAS placenta tissues. In addition, we also conducted IHC to validate the differential abundance of HSPA4. As shown in Figure 4, consistent with western blot assay, HSPA4 kept a significant higher level in PAS samples. In summary, for HSPA4 and CSH, the results of western blot assays were consistent with those of 2D-DIGE assays. Therefore, HSPA4 and CSH can serve as protein biomarkers of PAS.

### 2.4. HSPA4 Overexpression Enhanced the Angiogenesis Ability of Vessel Endothelial Cells

**Figure 5:**
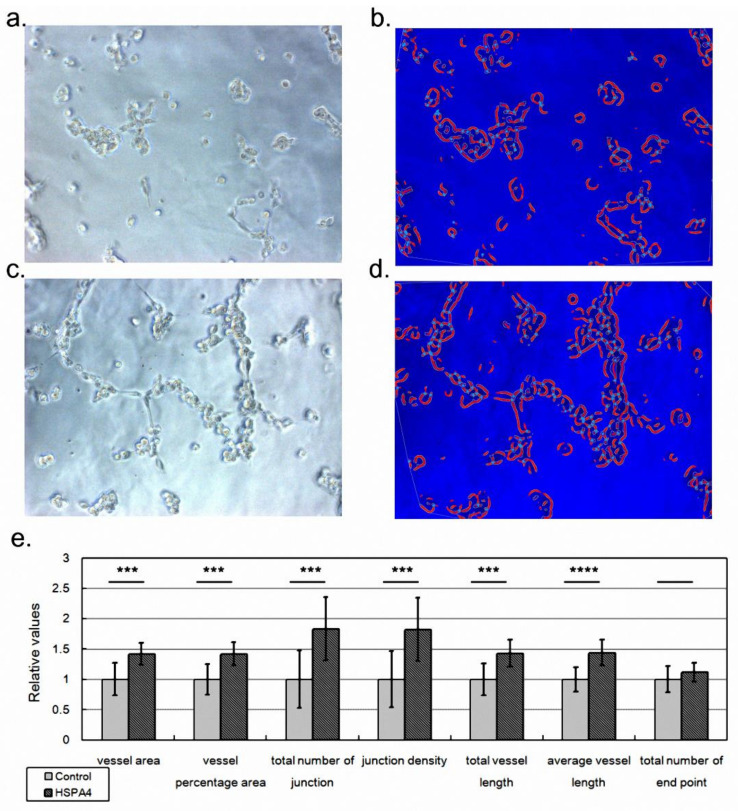
The results of the tube formation assay. We applied a tube formation assay to investigate whether HSPA4 promoted the angiogenesis ability of HUVECs. The cell growth morphologies of HUVECs transfected with empty pcDNA3.1 construct (**a**, control set) or with HSPA4 expression construct (**b**, HSPA4 set). (**c**,**d**) AngioTool analyzed the cell growth morphology and marked the vessels (the thick red lines), highlighted the junctions (the light blue points), and depicted the outlines of vessels (the thin orange lines). (**e**) By analyzing 12 pictures from four independent assays (four independent assays/wells * three pictures), we evaluated the angiogenesis ability by comparing these values. For simplicity, the values in the control set were normalized as one. Data was presented as the mean ± S.D. *** and **** denoted *p*-value < 0.001 and *p*-value < 0.0001, respectively.

PAS is characterized by uncontrolled growth of placenta tissue into the muscular layer of the uterus. Therefore, the angiogenesis ability of placenta tissue could be over-activated to promote placenta invasion [14]. Among the identified protein biomarkers of PAS, we selected HSPA4 for further functional assays since it was implied to be associated with tumor metastasis [15,16], a biological activity for which angiogenesis is usually enhanced. We hypothesized that the increased level of HSPA4 protein enhanced the angiogenesis ability of placenta vessels so that invasive placenta accreta was formed. To examine this hypothesis, we overexpressed HSPA4 in HUVECs followed by a tube formation assay as described in a previous study [17]. As expected, HSPA4 overexpression enhanced the mRNA level of HSPA4 by approximately 2-fold compared with the control (*p* = 0.002 by three independent assays). As shown in Figure 5, compared with control (Figure 5a,b), HSPA4 overexpression promoted HUVEC growth (Figure 5c,d). AngioTool also showed that HSPA4 overexpression (Figure 5d) caused HUVECs to form more tubes (the red line) and more junctions (the light blue dots) than the control (Figure 5b). By four independent assays, HSPA4 overexpression significantly overwhelmed the control in terms of most indices, including vessel area, vessel percentage area, total number of junctions, junction density, total vessel length, and average vessel length (Figure 5e). This result proved our hypothesis that HSPA4 promoted the angiogenesis ability of the placenta and could therefore lead to placenta accreta.

## 3. Discussion

In summary, our study not only identified the protein biomarkers of PAS but also demonstrated that HSPA4 enhanced the angiogenesis ability of vessel endothelial cells, which could explain the pathogenesis mechanism of HSPA4 in PAS. Placenta accreta spectrum (PAS) may cause severe blood loss during delivery so that it is the major lethal cause of obstetric hemorrhage [18,19]. For several decades, investigators have attempted to identify biochemical and/or biological markers that could be used to improve the accuracy of the antenatal diagnosis of placenta accreta. In this study, by comparing normal pregnancy (NP) and PAS placenta tissues, we identified 19 proteins differentially abundant between NP and PAS placenta tissues. Further western blot assays confirmed two protein biomarkers of PAS, including HSPA4 and CSH, which highlighted the robustness and reliability of the initially applied 2D-DIGE assay.

Heat shock protein family A (Hsp70) member 4 (HSPA4) belongs to the heat shock protein gene family. Our understanding on the relationship between HSPA4 and obstetrics-related disease is limited. Since PAS is characterized with uncontrolled growth of placenta tissue owing to its enhanced angiogenesis activity, we investigated whether HSPA4 could contribute to the pathogenesis mechanism of PAS by literature search. Wu et al. found that knock-down of HSPA4 decreased the in vitro migration and invasion activities of H1299 cells [15]. Zhong and colleagues also concluded that silencing HSPA4 inhibited the cell growth HT29 cells via increasing their apoptosis activity [16]. In addition, a previous study detected higher mRNA and protein levels of HSPA4 gene in the cancer stem cells with advanced invasion depth, lymph node metastasis or distant metastasis [20]. These studies implied that HSPA4 was involved in promoting angiogenesis activity which could lead to the invasion of trophoblast villous in PAS.

Chorionic somatomammotropin hormone (CSH), also known as human placental lactogen (hPL), is one type of polypeptide placental hormone with the ability to modify the metabolic state of pregnant women to facilitate the energy supply of the fetus [21]. CSH was identified as a biomarker of maternal obesity, diabetes and fetal growth abnormalities. Abnormal CSH secretion in circulation was associated with an increased risk of gestational complications, such as placental dysfunction, diabetic retinopathy, and abnormalities in fetal growth [22]. In addition to regulating the maternal metabolic state, CSH was also involved in the regulation of angiogenesis [23].

To date, the patients suffering PAS are usually recommended to undertake a cesarean delivery. Cesarean hysterectomy is currently considered the preferred method in most national societies [18]. However cesarean hysterectomy sacrifices fertility in young women and possibly causes maternal morbidity and mortality. Conservative, uterine-sparing management accompanied by arterial occlusion has been reported to be successful [24]. Hsu et al. reported in 2020 that planned conservative management with prophylactic transcatheter arterial embolization (TAE) and leaving placenta in situ is feasible and safe for 19 of 23 women with placenta increta or percreta who desire fertility preservation, 4 patients (19%) underwent hysterectomy due to secondary postpartum hemorrhage [25]. Since the promoted angiogenesis by HSPA4 in the invading placenta tissue could explain the pathogenetic mechanism of PAS, it deserves further attention to investigate whether HSPA4 is a possible therapeutic target of PAS. In other words, it is possible to use an animal model to investigate whether specifically repressing HSPA4 could attenuate PAS in vivo.

In this study, we identified the PAS biomarker in placenta tissues. To make HSPA4 an applicable biomarker of PAS, it is necessary to investigate whether the variation of HSAP4 protein can be observed in serum samples. After all, a serum biomarker is more applicable than a tissue biomarker in detecting disease. PAS results from the uncontrolled growth of the placenta. Therefore, the examinations of angiogenesis ability should be conducted on the endothelial cells of placenta vessels. However, the endothelial cells of placenta vessels were not available. Without the volunteer donor, the commercial company could not provide us with this cell line. Therefore, we used HUVEC, a widely used vessel endothelial cell, instead. In this study, this is of course a disadvantage but also an unavoidable alternative. In addition to identifying PAS protein biomarkers, we also connected the promoted angiogenesis of vessel endothelial cells with placenta invasion, investigating the pathogenetic mechanism of PAS.

## 4. Materials and Methods

### 4.1. Subject Enrollment and Sample Collection

This study was approved by the institutional ethics board of Kaohsiung Chang Gung Memorial Hospital (IRB number: 96-1803B and 100-4466A3). Two well-trained pathologists were responsible for collecting and dissecting the placenta tissues. Five human placenta tissues were collected from the pregnant subjects who were complicated with PAS and underwent hysterectomy after delivery. Another five placenta tissues were collected from the subjects with normal pregnancy (NP). To compare the protein profiles and to identify the PAS-associated proteins, for the PAS placenta tissues, we specifically isolated the trophoblast villous invading into the uterine muscle layer. For the NP placenta tissues, their corresponding compartments were isolated. These collected tissue samples were stored either as formalin-fixed paraffin-embedded (FFPE) blocks or as frozen fresh samples at −80 °C. Before assays, these FFPE blocks or frozen fresh samples were examined by pathologists again to ensure that trophoblast villous tissues were collected for further assays.

### 4.2. Protein Enrichment from Cell Lysate

To enrich proteins from placenta tissues, the tissue pieces from 10 frozen fresh samples (for the 10 individual subjects) were placed in a 2-mL microcentrifuge tube with DIGE lysis buffer added. Then, we used a mechanical homogenizer to breakdown the cells, followed by centrifugation at 14,000 rpm for 10 min at 4 °C. The supernatants were collected and desalted by using Vivaspin 500 with a 3000 molecular weight cut off. Finally, protein concentration was determined using a 2-D Quant Kit.

### 4.3. Protein Quantification with 2D-DIGE and Identification with MALDI TOF

In this study, we used a two-dimensional differential in-gel electrophoresis (2D-DIGE) assay to quantify proteins, followed by a MALDI TOF/TOF assay to identify proteins by referring to our previous studies [26,27]. In summary, after being prepared with the 2-D DIGE Kit (GE Healthcare Bio-Sciences, North Brunswick, NJ, USA), the PAS and NP protein samples were labeled with Cy3 and Cy5 dye, respectively. With Cy2 applied as the reference pool, the labeled proteins were multiplexed and resolved in one gel, followed by scanning and quantification. The spots with an empirical abundance ratio ≥1.5 (the average from five pairs of PAS vs. five NP comparisons) were considered to be differentially abundant. After scanning, the five gels were stained with ammoniacal silver nitrate solution and the spots selected based on 2D-DIGE assays were further selected for in-gel digestion and MALDI TOF/TOF (Bruker, Bremen, Germany). By using MASCOT software (Matrix Science, Boston, MA, USA) referring to the SwissProt and NCBInr databases, a peptide/protein can be identified.

### 4.4. Western Blot Assay

Equal amounts of protein from placenta tissues were separated by 10%,12%, and 15% SDS-PAGE, under reducing conditions. After electrophoresis, the proteins were electrotransferred to Hybond-P PVDF transfer membranes (GE Healthcare Bio-Sciences, Buckinghamshire, UK), and blots were blocked for 1 h at room temperature with 5% nonfat milk in TBST. The membranes were then incubated overnight at 4 °C with the appropriate dilution of rabbit polyclonal antibody to insulin-like growth factor binding protein 1 (IGFBP-1) (Abcam, Cambridge, UK), rabbit monoclonal antibody to transthyretin (Epitomics, Burlingame, CA, USA) and mouse monoclonal antibody to alpha 1 antitrypsin (Abcam, Cambridge, UK) in blocking buffer. After being washed, membranes were incubated with goat anti-rabbit IgG-HRP (Santa Cruz Biotechnology, Santa Cruz, CA, USA) and goat anti-mouse IgG-HRP (Santa Cruz Biotechnology, Santa Cruz, CA, USA) as secondary antibodies. Bound antibody was detected by the ECL plus western blotting detection system (GE Healthcare Bio-Sciences, Buckinghamshire, UK). Equal protein loading was confirmed by exposure of the membranes to mouse monoclonal IgG (Santa Cruz Biotechnology, Santa Cruz, CA, USA). Western blots were scanned and images were quantified for protein content using ImageJ software. Mean protein quantification after western blot analysis was performed by three independent experiments.

### 4.5. Immunohistochemistry Assay

Placenta tissues were first fixed with 10% formalin and further subjected to dehydration and paraffin embedment. Then, 3μm thick tissue sections were collected and deparaffinized in xylene for 3 runs and 5 min for each run. Next, tissue sections were incubated in Ultra Vision Hydrogen Peroxide Block Solution (BIOCHEMICALTM; HPBS-191001, Hao-Long Biology Technology Inc., Kaohsiung, Taiwan) at room temperature for 10 min to block endogenous peroxidase activity, followed by a PBS rinse for 2 runs and 5 min for each run. To determine the abundance of HSPA4 protein, we performed antigen retrieval to unmask the antigenic epitope by using Epitope Retrieval Solution (BIOCHEMICALTM; ERS-191001, Hao-Long Biology Technology Inc., Kaohsiung, Taiwan). We then removed the staining container to room temperature and allowed the slides to cool for 20 min, followed by a PBS rinse for 2 runs and 5 min for each run. Finally, tissue sections were applied with 100 μL diluted primary HSPA4 antibody (1:200, Cat. No. HPA010023, MERK) and incubated at 4 °C, overnight, followed by a PBS wash. Next, the tissue sections were in order subjected to incubation with 100 μL appropriately ImmPRESSTM HRP REAGENT KIT (Cat. No. MP-7500, VECTOR) at room temperature for 30 min and with ImmPACTTMDAB (Cat. No. SK-4105, VECTOR) for 3 min. Tissue sections were further counterstained by immersing sides in Hematoxylin for 1 min, followed by running tap water rinse for 10 min. Finally, tissue sections were dehydrated through 4 times of alcohol (95%, 95%, 100% and 100%), 5 min each. The tissue slides in xylene and coverslip were cleared using mounting solution. The mounted slides can be stored at room temperature permanently. 

### 4.6. Enhancing HSPA4 Expression with Construct

To enhance the expression of HSPA4, we first obtained the overexpression construct of the HSPA4 gene (PA1807G, Topgenbio, Kaohsiung, Taiwan). This construct was generated by cloning the open reading frame of the HSPA4 gene into the pcDNA3.1 vector (PA1924T, Topgenbio, Kaohsiung, Taiwan). Then, we transfected 0.5 μg of HSPA4 construct into HUVECs (C2517A, Lonza, Cohasset, MN, USA) with Lipofectamine 2000 transfection reagent (11668030, Thermo, Waltham, MA, USA). In addition, for comparison, 0.5 μg of empty pcDNA3.1 construct was also transfected into another plate of HUVECs.

### 4.7. Evaluating the Angiogenesis Ability of HUVECs with Tube Formation Assay

We used a tube formation assay kit (K905, BioVision, Milpitas, CA, USA) to evaluate the angiogenesis ability of HUVECs. In summary, we first prepared the wells by referring to the manufacturer’s instructions. Then, 1*104 HUVECs transfected with either HSPA4 construct or empty pcDNA3.1 construct were seeded into the well for three hours. Then, for each well, we randomly took three pictures with 200× amplification to record the cell growth morphology.

### 4.8. Analyzing Cell Growth Morphology with AngioTool

The pictures recording cell growth morphology were analyzed with AngioTool, a toolkit developed specifically for analyzing the angiogenesis patterns of endothelial cells [28]. AngioTool first determined the explant area of the analyzed picture, followed by marking the outlines of the vessel (tube formed by endothelial cells) and the locations of junctions. As a result, this information was quantified and reported. To exclude the background noise, two parameters were specified as follows: “Vessel intensity” was set from 60 to 240 and the “Remove small particles” option was selected.

## Figures and Tables

**Table 1:** Demographic table. We enrolled 10 pregnant subjects to donate their placenta tissues after delivery. The details of these 10 subjects are tabulated below. *p*-value was calculated based on a *t*-test.

	Normal Pregnancy (NP, *n* = 5)	Placenta Accreta Spectrum (PAS, *n* = 5)	*p*-Value
Age (years old)	31 ± 3.9	33.6 ± 1.2	NS
GA (week)	36 ± 1.1	35.8 ± 2.3	NS
NW (g)	2940 ± 224.9	2684 ± 514.6	NS

GA, gestational age; NW, newborn weight; Data are expressed as the mean ± S.D. NS: non-significant.

**Table 2:** Clinical characteristics of the subjects with placenta accreta complications. All PAS subjects underwent hysterectomy after delivery.

Case	Age	Gestational Age	Type of Placenta Accreta	Blood Loss (mL)
1	32	37	Percreta	6000
2	33	38	Increta	14,110
3	33	33	Percreta	4500
4	35	33	Increta	1900
5	35	38	Increta	1500

**Table 3:** Details of the differentially abundant proteins identified between five NP and five PAS placenta tissues. We used 2D-DIGE and MALDI TOF/TOF to quantify and identify proteins, respectively. The theoretical pI/Mr, sequence coverage and score were determined by the MALDI TOP/TOF assay. The fold change and *p*-value were calculate by the 2D-DIGE assay. Positive fold change denoted a higher abundance in PAS samples. *p*-value was calculated based on a *t*-test.

Spot No.	SwissPort Entry Name	Protein Name	Theoretical *pI/Mr*	Sequence Coverage, MS%	Score	Fold Change	*p*-Value
1	A2KLM6	Immunoglobulin heavy chain variable region	5.25/11	77	68	−1.69	0.052
2	HSPA4	Heat shock 70 kDa protein 4	5.11/94.2	20	57	4.92	0.045
3	PRKAR2	cAMP-dependent protein kinase type II-alpha regulatory subunit	4.96/45.4	33	68	7.42	0.023
4	GSTP1	Glutathione S-transferase P	5.43/23.3	47	66	−1.50	0.054
5	MYNN	Myoneurin	5.72/10.6	79	69	−1.66	0.038
6	ZN211	Zinc finger protein 211	8.83/64.4	27	56	−1.65	0.041
7	UTS2	Urotensin-2 isoform a preproprotein	7/16	41	66	2.00	0.0052
8	ZN157	Zinc finger protein 157	8.83/58.2	29	62	1.98	0.025
9	ACTB	Actin, cytoplasmic 1	5.29/41.7	25	58	5.36	0.021
10	CSH	Chorionic somatomammotropin hormone	5.34/25	57	147	−1.63	0.028
11	IQEC1	IQ motif and SEC7 domain-containing protein 1	6.49/108	17	58	2.54	0.053
12	CLIC1	Chloride intracellular channel protein 1	5.09/26.9	51	140	1.54	0.037
13	PMFBP	Polyamine-modulated factor 1-binding protein 1	5.94/118	22	59	1.91	0.010
14	DDX4	Probable ATP-dependent RNA helicase DDX4	5.62/79.2	20	57	−1.51	0.014
15	PLXB2	Plexin-B2	5.85/204	15	58	−1.63	0.044
16	D3DT17	hCG2038441	9.28/16.3	65	67	1.59	0.035
17	B7ZC06	Golgi autoantigen, golgin subfamily a, 2	6.05/53.3	20	67	1.62	0.0032
18	AMBP	Protein AMBP	5.95/38.9	38	56	−1.67	0.025
19	ANXA2	Annexin A2	7.57/38.5	28	86	−1.56	0.0060

## Data Availability

Not applicable.

## References

[1] Wu S., Kocherginsky M., Hibbard J.U. (2005). Abnormal placentation: Twenty-year analysis. Am. J. Obstet. Gynecol..

[2] Yu P.C., Ou H.Y., Tsang L.L., Kung F.T., Hsu T.Y., Cheng Y.F. (2009). Prophylactic intraoperative uterine artery embolization to control hemorrhage in abnormal placentation during late gestation. Fertil. Steril..

[3] Tantbirojn P., Crum C.P., Parast M.M. (2008). Pathophysiology of placenta creta: The role of decidua and extravillous trophoblast. Placenta.

[4] Silver R.M., Landon M.B., Rouse D.J., Leveno K.J., Spong C.Y., Thom E.A., Moawad A.H., Caritis S.N., Harper M., Wapner R.J. (2006). Maternal morbidity associated with multiple repeat cesarean deliveries. Obstet. Gynecol..

[5] Miller D.A., Chollet J.A., Goodwin T.M. (1997). Clinical risk factors for placenta previa-placenta accreta. Am. J. Obstet. Gynecol..

[6] Chou M.M., Ho E.S., Lee Y.H. (2000). Prenatal diagnosis of placenta previa accreta by transabdominal color Doppler ultrasound. Ultrasound Obstet. Gynecol..

[7] Thorp J.M., Councell R.B., Sandridge D.A., Wiest H.H. (1992). Antepartum diagnosis of placenta previa percreta by magnetic resonance imaging. Obstet. Gynecol..

[8] Du M.R., Zhou W.H., Yan F.T., Zhu X.Y., He Y.Y., Yang J.Y., Li D.J. (2007). Cyclosporine A induces titin expression via MAPK/ERK signalling and improves proliferative and invasive potential of human trophoblast cells. Hum. Reprod..

[9] Staun-Ram E., Goldman S., Shalev E. (2009). Ets-2 and p53 mediate cAMP-induced MMP-2 expression, activity and trophoblast invasion. Reprod. Biol. Endocrinol..

[10] Tseng J.J., Hsu S.L., Wen M.C., Ho E.S., Chou M.M. (2004). Expression of epidermal growth factor receptor and c-erbB-2 oncoprotein in trophoblast populations of placenta accreta. Am. J. Obstet. Gynecol..

[11] Goh W., Yamamoto S.Y., Thompson K.S., Bryant-Greenwood G.D. (2013). Relaxin, Its Receptor (RXFP1), and Insulin-Like Peptide 4 Expression Through Gestation and in Placenta Accreta. Reprod. Sci..

[12] Duncan M.W., Hunsucker S.W. (2005). Proteomics as a tool for clinically relevant biomarker discovery and validation. Exp. Biol. Med..

[13] Marko-Varga G., Fehniger T.E. (2004). Proteomics and disease—The challenges for technology and discovery. J. Proteome Res..

[14] Bartels H.C., Postle J.D., Downey P., Brennan D.J. (2018). Placenta Accreta Spectrum: A Review of Pathology, Molecular Biology, and Biomarkers. Dis. Markers.

[15] Wu C.Y., Lin C.T., Wu M.Z., Wu K.J. (2011). Induction of HSPA4 and HSPA14 by NBS1 overexpression contributes to NBS1-induced in vitro metastatic and transformation activity. J. Biomed. Sci..

[16] Zhong M.A., Zhang H., Qi X.Y., Lu A.G., You T.G., Gao W., Guo X.L., Zhou Z.Q., Yang Y., Wang C.J. (2011). ShRNA-mediated gene silencing of heat shock protein 70 inhibits human colon cancer growth. Mol. Med. Rep..

[17] Lee F.Y., Sun C.K., Sung P.H., Chen K.H., Chua S., Sheu J.J., Chung S.Y., Chai H.T., Chen Y.L., Huang T.H. (2018). Daily melatonin protects the endothelial lineage and functional integrity against the aging process, oxidative stress, and toxic environment and restores blood flow in critical limb ischemia area in mice. J. Pineal Res..

[18] Einerson B.D., Branch D.W. (2018). Surgical Management of Placenta Accreta Spectrum. Clin. Obstet. Gynecol..

[19] Silver R.M., Branch D.W. (2018). Placenta Accreta Spectrum. N. Engl. J. Med..

[20] Morisaki T., Yashiro M., Kakehashi A., Inagaki A., Kinoshita H., Fukuoka T., Kasashima H., Masuda G., Sakurai K., Kubo N. (2014). Comparative proteomics analysis of gastric cancer stem cells. PLoS ONE.

[21] Josimovich J.B., Atwood B.L., Goss D.A. (1963). Luteotrophic, Immunologic and Electrophoretic Properties of Human Placental Lactogen. Endocrinology.

[22] Sibiak R., Jankowski M., Gutaj P., Mozdziak P., Kempisty B., Wender-Ożegowska E. (2020). Placental Lactogen as a Marker of Maternal Obesity, Diabetes, and Fetal Growth Abnormalities: Current Knowledge and Clinical Perspectives. J. Clin. Med..

[23] Corbacho A.M., Martinez De La Escalera G., Clapp C. (2002). Roles of prolactin and related members of the prolactin/growth hormone/placental lactogen family in angiogenesis. J. Endocrinol..

[24] Lo T.K., Yung W.K., Lau W.L., Law B., Lau S., Leung W.C. (2014). Planned conservative management of placenta accrete—Experience of a regional general hospital. J. Matern.-Fetal Neonatal Med..

[25] Huang K.L., Leung-Chit Tsang L., Cheng Y.F., Huang F.J., Fu H.C., Kung F.T., Tsai C.C., Cheng H.H., Lai Y.J., Ou C.Y. (2020). Planned conservative management of placenta increta and percreta with prophylactic transcatheter arterial embolization and leaving placenta in situ for women who desire fertility preservation. Placenta.

[26] Hsu T.Y., Lin H., Hung H.N., Yang K.D., Ou C.Y., Tsai C.C., Cheng H.H., Chung S.H., Cheng B.H., Wong Y.H. (2016). Two-Dimensional Differential Gel Electrophoresis to Identify Protein Biomarkers in Amniotic Fluid of Edwards Syndrome (Trisomy 18) Pregnancies. PLoS ONE.

[27] Hsu T.Y., Tsai K.W., Lan K.C., Hung H.N., Lai Y.J., Cheng H.H., Tsai C.C., Li S.C. (2020). Identifying the potential protein biomarkers of preterm birth in amniotic fluid. Taiwan J. Obstet. Gynecol..

[28] Zudaire E., Gambardella L., Kurcz C., Vermeren S. (2011). A computational tool for quantitative analysis of vascular networks. PLoS ONE.

